# Assessment of patient specific information in the wild on fundus photography and optical coherence tomography

**DOI:** 10.1038/s41598-021-86577-5

**Published:** 2021-04-21

**Authors:** Marion R. Munk, Thomas Kurmann, Pablo Márquez-Neila, Martin S. Zinkernagel, Sebastian Wolf, Raphael Sznitman

**Affiliations:** 1 Department of Ophthalmology, Inselspital, Bern University Hospital, Bern, Switzerland; 2 Bern Photographic Reading Center (BPRC), Bern University Hospital, Inselspital, Bern, Switzerland; 3 ARTORG Center, University of Bern, Bern, Switzerland

**Keywords:** Medical research, Computer science

## Abstract

In this paper we analyse the performance of machine learning methods in predicting patient information such as age or sex solely from retinal imaging modalities in a heterogeneous clinical population. Our dataset consists of N = 135,667 fundus images and N = 85,536 volumetric OCT scans. Deep learning models were trained to predict the patient’s age and sex from fundus images, OCT cross sections and OCT volumes. For sex prediction, a ROC AUC of 0.80 was achieved for fundus images, 0.84 for OCT cross sections and 0.90 for OCT volumes. Age prediction mean absolute errors of 6.328 years for fundus, 5.625 years for OCT cross sections and 4.541 for OCT volumes were observed. We assess the performance of OCT scans containing different biomarkers and note a peak performance of AUC = 0.88 for OCT cross sections and 0.95 for volumes when there is no pathology on scans. Performance drops in case of drusen, fibrovascular pigment epitheliuum detachment and geographic atrophy present. We conclude that deep learning based methods are capable of classifying the patient’s sex and age from color fundus photography and OCT for a broad spectrum of patients irrespective of underlying disease or image quality. Non-random sex prediction using fundus images seems only possible if the eye fovea and optic disc are visible.

## Introduction

Machine learning has seen widespread adoption in ophthalmology^1^. It is employed to detect and differentiate retinal diseases and glaucoma^2–4^, stratify risk for disease onset and predict treatment response^5, 6^. It is also applied to automatically assess image quality, to quantify and segment retinal structures and to improve image quality of color fundus images, optical coherence tomography (OCT) and OCT angiography^7–9^. A large focus of previous work has been in performing tasks which may augment or substitute human experts. These tasks typically require transferring knowledge from domain experts, in the form of annotations and their respective images, to a machine learning process, whereby implying that a human is capable of doing the task. However, machine learning can also be used in an exploratory manner, to extract information and patterns which are not obvious to the human eye. For instance, cardiovascular risk factors such as age, smoking status and systolic blood pressure were successfully predicted using color fundus images in large diabetic patient cohort^10^. However, this study only considered a distinct patient population and only a unique image modality type. Additional studies^11, 12^ have been performed to analyse the effects of age and sex on the prediction of pathologies and vice-versa. The main focus of these studies has been on fundus imaging, whereas other imaging modalities such as OCT provide feature rich images of the layers of the retina. Given the large spectrum of diseases typically found in a clinical setting, we investigate if machine learning based models are capable of extracting the gender and age from a patient’s fundus image or OCT image data regardless of any ocular disease or severity.

## Method

### Datasets

Datasets from two different retinal imaging modalities, namely fundus photography and OCT, were collected at the Department of Ophthalmology, University clinic Bern (Bern, Switzerland). The fundus photography dataset consists of 135,667 images of 16,196, patients (8180 female, 8016 male; mean age = 57.767, $\sigma =20.72$). 69,002 images are from male patients and 66,665 from female patients. Fundus images included color fundus photography as well as red free images independent of field of view of the entire variety of diseases seen in a tertiary eye center. All images, regardless of image quality, were included. However, no wide field or ultrawide field images were included in this analysis.

The OCT dataset contained a total of 85,536 Heidelberg Spectralis OCT volume-or CScans (Spectralis, Heidelberg Engineering, Germany) from 5578 patients (2694 female, 2884 male, mean age = 65.3466, $\sigma =17.94$) seen at the Department of Ophthalmology, University clinic Bern. 45,048 scans are from male patients and 40,488 female patients. Scans cover a $6 \times 6\;{\text {mm}}^{2}$ area centered on the macula and consisted of 49 single BScans, each nine times averaged. All images irrespective of quality and eye pathology were included. We include multiple scans from the same patient to reflect the disease and treatment history. This study was approved by the ethics committee of the Kanton of Bern, Switzerland (KEK-Nr. 2019-00285). The study is in compliance with the tenets of the Declaration of Helsinki. Given the retrospective design of this study, the ethics committee of the Kanton of Bern, Switzerland waived the requirement for individual informed consent.

### Classification of biological sex and age

In this section we describe our method to predict the biological sex and age of a patient from an image. We consider three cases types of images: fundus photography, OCT slices (BScans) and OCT volumes (CScans). Our target is a multi-task problem whereby the patient sex and age must be estimated. The former is cast as a classification problem while the latter is a regression problem. To do so, we use a Convolutional Neural Network (CNN) which is optimized to minimize the sum of the loss of each task.

The age prediction task could be formulated as a direct regression problem, where the output of the network is a scalar value corresponding to the age of the patient. This has the disadvantage that an inherent scale must be learned. Another approach would be to predict patient age bins using multi-class classification. Depending on the size of the bins, this could lead to low granularity or a large amount of classes with potentially few samples. Instead of these formulations, we propose a mixture of both methods, where the network outputs age bins (n = 11, delta = 10 years), which are normalized using a softmax activation $\omega$ and multiplied by the bins lower edge $d_x$. Finally, the sum of all bins are computed and compared to the ground truth value using the L2-norm. For the binary sex classification we use the standard binary cross-entropy. We weigh the individual task loss functions, since the loss scales are not comparable and without would bias one task over the other. The weight $w_{sex}$ is set to 1 and $w_{age}$ dynamically updated to keep the scale of $L_{sex}$ and $L_{age}$ equivalent

1 $$\begin{aligned} L(y, \hat{y})&= w_{sex} L_{sex}(y_{sex}, {{\hat {y}}_{sex}}) + w_{age} L_{age}(y_{age}, {{\hat {y}}_{age}}) \end{aligned}$$

2 $$\begin{aligned} L_{age}(y,\hat{y})&= \left( y - \sum _x \omega (\hat{y})_x * d_x\right) ^2. \end{aligned}$$

Given the comparably large amount of data in the datasets, we choose to use the same ResNet-152^13^ CNN architecture for all experiments. The prediction of OCT CScans require a further step as the modality is volumetric in contrast to fundus images and OCT BScans. Every BScan of a CScan volume is passed through the trained CNN and the feature vector of size [$1 \times 4096 \times 49 \times 1$] is extracted, which is then compressed using a 2d convolution [$1 \times 512 \times 1 \times 1$] and fused to generate the final prediction [$1 \times 12$]. We reuse the trained BScan network for the CNN to leverage already learnt features. Our C-Scan prediction architecture is depicted in Fig. 3. For OCT BScans and Fundus images, we directly predict the sex and age without additional fusion.

**Figure 1:**
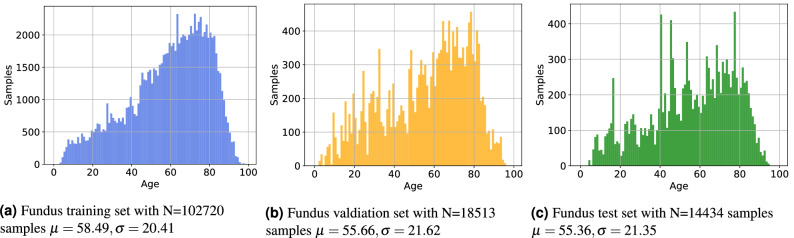
Histogram of patient ages for the corresponding the fundus data sets.

**Figure 2:**
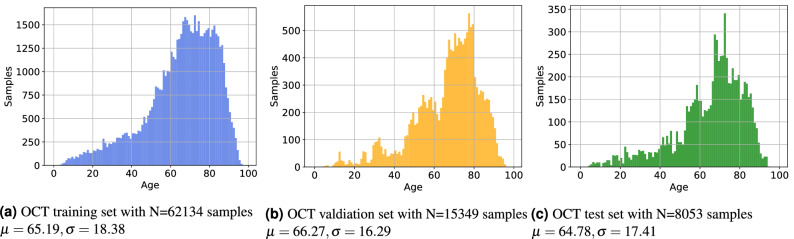
Histogram of patient ages for the corresponding the OCT data sets.

For all experiments we split the data into 80% training, 10% validation and 10% testing. In order to avoid biasing predictions, we do not perform an image-wise split, but rather split the data patient-wise. We use the same split for the OCT B-Scan and C-Scan experiments. Table 1 shows the statistics of the datasets. In Figs. 1 and 2 we show the histograms of the data splits, illustrating that the distribution of all splits are similar to one another.

**Table 1:** Dataset statistics.

	Fundus	OCT BScan	OCT CScan
**Training**			
Samples	102,720 (0.50/0.50)	3,044,566 (0.470/0.530)	62,134 (0.470/0.530)
Patients	13,113 (0.51/0.49)	4519 (0.482/0.518)	4519 (0.482/0.518)
Mean age	58.49 ($\sigma =20.41$)	65.19 ($\sigma =18.38$)	65.19 ($\sigma =18.38$)
**Validation**			
Samples	18,513 (0.47/0.53)	752,101 (0.486/0.514)	15,349 (0.486/0.514)
Patients	1457 (0.50/0.50)	502 (0.498/0.502)	502 (0.498/0.502)
Mean age	55.66 ($\sigma =21.62$)	66.27 ($\sigma =16.28$)	66.27 ($\sigma =16.28$)
**Test**			
Samples	14,434 (0.46/0.54)	394,597 (0.472/0.528)	8053 (0.472/0.528)
Patients	1618 (0.504/0.495)	557 (0.474/0.526)	557 (0.474/0.526)
Mean age	55.36 ($\sigma =21.347$)	64.78 ($\sigma =17.414$)	64.78 ($\sigma =17.414$)
**Total**			
Samples	135,667 (0.49/0.51)	4,191,264 (0.473/0.527)	85,536 (0.473/0.527)
Patients	16,188 (0.505/0.495)	5578 (0.483/0.5127)	5578 (0.483/0.5127)
Mean age	57.77 ($\sigma =20.72$)	65.34 ($\sigma =17.94$)	65.34 ($\sigma =17.94$)

Values inside brackets denote the female/male ratio of the set.

**Figure 3:**
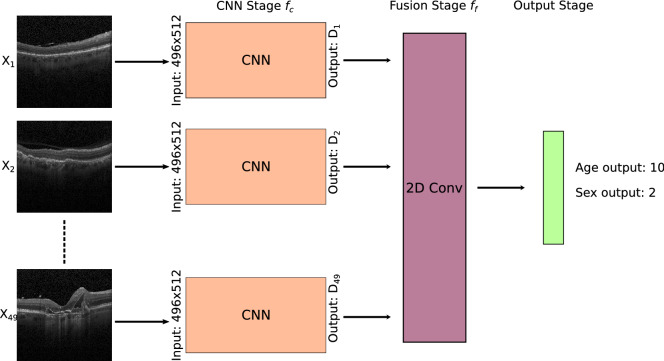
Overview of the CScan method. Features of every independant BScan are extracted using the CNN stage $f_c$. The features are fused using a 2D convolution and classified using a fully connected layers.

We train all networks using the Adam optimizer^14^ with an initial learning rate of 1e − 4, which is reduced after 15 and 30 epochs by a factor of 10. We use L2 weight regularization (1e − 5) and dropout (p = 0.25) in the last layer. We correct the age imbalance in the datasets by oversampling cases according to the inverse sample probability. To do so, we first approximate the age distribution using a histogram with a fixed bin size of 10 years. The probability of a sample *x* to be chosen during training is then $p(x) = {\frac{w_x}{N}}$, where N is the total amount of samples in the dataset and $w_x = {\frac{1}{p_{age}}(x_{age})}$ (Fig. 3).

## Results

### Sex and age prediction on OCT and fundus images

**Figure 4:**
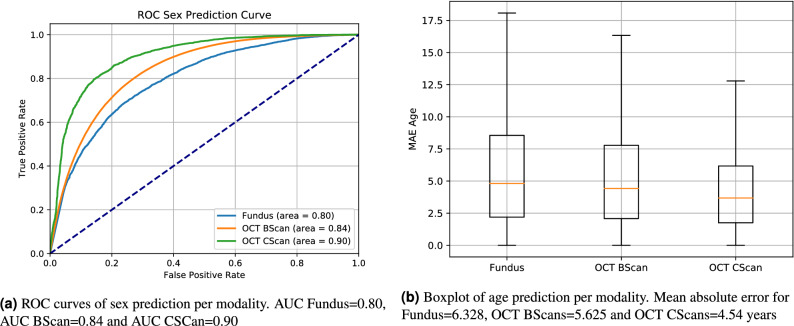
Results of automated age and sex prediction compared by modality.

The results of the sex prediction for all three modalities is shown in Fig. 4. We observe an AUC ROC of 0.80 for fundus images, 0.84 for OCT BScans and 0.90 for OCT CScans. Thresholding the prediction results in an accuracy of 0.73 for fundus, 0.76 for OCT BScans and 0.83 for CScans. Results of the age prediction are evaluated using the mean absolute error (MAE) and shown in Fig. 4b. Using fundus imaging, we observe MAE = 6.328 $\sigma =5.928$, OCT BScan MAE = 5.625 $\sigma =4.898$ and OCT CScan MAE = 4.541 $\sigma =3.909$. We test the age predictions for significant difference using Welchs t-test and report ${\text {p}}<0.001$ for OCT CScan vs fundus and OCT CScan vs OCT Bscan. Thus, the OCT CScans provided the best age prediction, followed by single OCT BScans and Fundus images.

**Figure 5:**
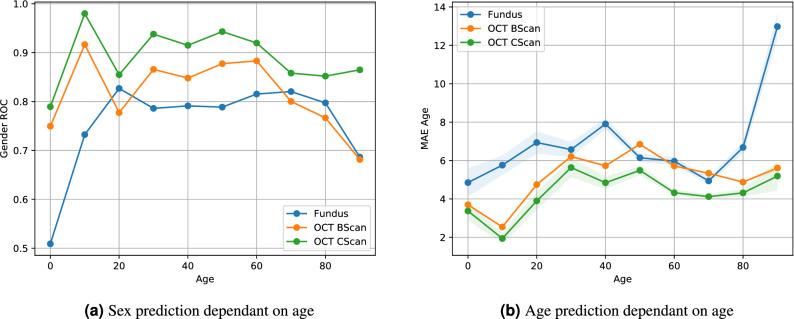
Age dependant results for sex and age binned into bins of 10 years. Independent of image modalitiy sex prediction is more accurate among age groups of $\leq 60$ years. Above the age of 60 the performance declines. Confidence intervals are provided for the right plot.

**Figure 6:**
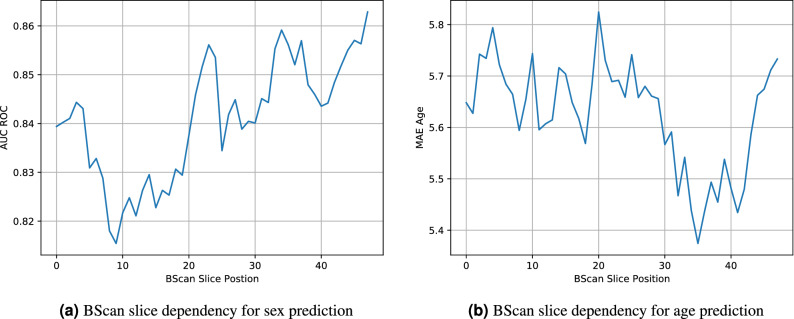
Analysis of the dependency of BScan slice position in CScans and resulting performance. Central foveal scans show a more precise prediction of sex and age, respectively.

In Fig. 5a, we show the age dependency of the sex prediction. We observe a noticeable reduction in performance over the age of 60, despite this age group making up the majority of the dataset. We observe the lowest age error for patients younger than 30 years of age for all modalities as shown in Fig. 5b. In Fig. 6, we analyse the dependency of a BScan position within a CScan for both sex and age prediction. We observe an increase in sex prediction performance towards the central foveal scans. For age prediction, the highest prediction performance lies in the same region between scans 30 and 40.

### Assessment of activation maps

To gain an understanding of the factors which differentiate sex and age of patients, we analyse the activation maps^15^ of the fundus image and OCT BScan predictions. In Fig. 8, we display correct and incorrect sex classification cases for OCT BScans. The OCTs attention maps highlight primarily the choriocapillaries and the whole choroid. Incorrect predictions were primarily seen in cases with impaired choroidal structures, invisible choroid and damaged outer retina with thinned and damaged choroid (see Fig. 8e–h).

For the case of fundus images, the region of the optic disc (OD), the macula and larger vessels within the posterior pole were highlighted (Fig. 9). Wrong predictions were seen in images, where respective parameters were invisible (Fig. 9). Activation maps of images with severe pathologies of the OD and macula tended to highlight pathological features rather than OD and macula for decision making (Fig. 9), which also impaired accuracy of correct prediction. Low image quality decreased performance as well. In order to assess the crucial role of the visibility of the optic disc and macula in more detail, we sampled randomly 260 predictions and binned them manually into four classes based on the visibility of the optic disc and macula. Assessing visibility and discriminability of the optic disc and the macula of on these led to 145 correct predictions and 61 incorrect sex predictions. In contrast, there were only 25 correct predictions when the optic disc and the macula was not visible against 29 incorrect sex predictions. Thus, non-random sex prediction was only possible if the macula and optic disc were visible and discriminable on fundus images.

### Assessment of biomarker influence

Our datasets contain a large cohort of patients with various pathologies. We analyse the effects of these pathologies on the performance of the age and sex prediction. While we do not have any information regarding clinical diagnosis of the patients and scans, we make use of an automated biomarker classifier for OCT scans which was shown to accurately detect biomarkers and classify pathologies^2^. This automated classifier predicts the presence of 11 biomarkers for every BScan at a human expert level^2^. This evaluation only concerns OCT scans.

**Figure 7:**
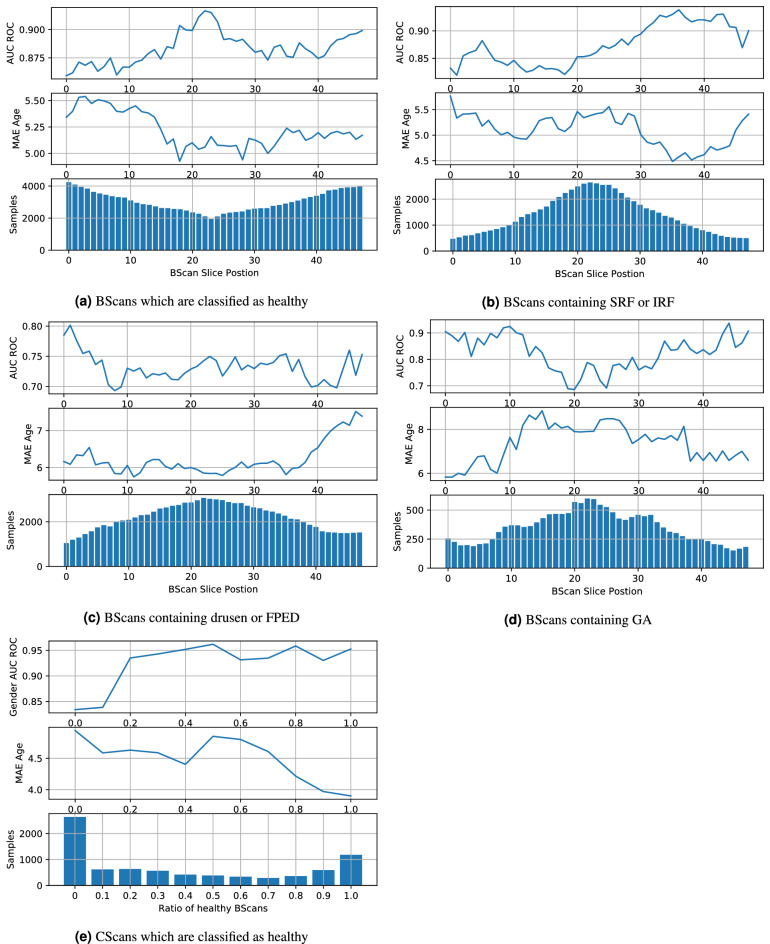
Analysis of the impact of biomarker presence in OCT BScans and CScans. Healthy scans show on average the best performance, whereas scans containing FPED or drusen show the lowest. The CScan performance for age and sex prediction is highest in scans with a 100% healthy ratio.

We extract all healthy OCT BScans (${\text {p}}>0.9$) from the test (N = 145,787) set and compute the age and sex prediction performance. We note an increase in sex prediction AUC = 0.882 (0.801 accuracy) and a decrease in age MAE = 5.238. We show the per slice results in Fig. 7a. On average, the maximum slice performance is found in the central scan region. We also evaluate the prediction performance in the presence of fluid (subretinal fluid, intraretinal fluid or intraretinal cysts). We extract all scans with fluid (${\text {p}}>0.9$) from the test set (N = 66,570). We observe AUC = 0.872 (0.777 accuracy) for the sex prediction and MAE = 5.145 for age prediction. Per slice results are shown in Fig. 7b. In addition, we perform the same evaluation on scans containing Drusen and Fibrovascular PED (N = 105,361 samples). We note a decrease in performance in sex prediction to an AUC = 0.730 (0.636 accuracy), and increase in MAE = 6.154. Per slice results are shown in Fig. 7c. Last, in the case of Geographic Atrophy (N = 16,762 samples), we observe a decrease in sex prediction to AUC = 0.8107 (0.752 accuracy) and an increase in age prediction error MAE = 7.592. Per slice results are shown in Fig. 7d. In general, when biomarkers are present, maximum slice performance is typically found in peripheral Bscans.

To analyse the dependency in OCT CScans, we extract all volumes which are healthy (all BScans ${\text {p}}>0.8$ healthy) leading to N = 1394 scans. Sex prediction performance increases to AUC = 0.946 (0.884 accuracy) while age prediction error decreases to MAE = 3.845. Results of different ratios of healthy BScans per CScan can be seen in Fig. 7e .

**Figure 8:**
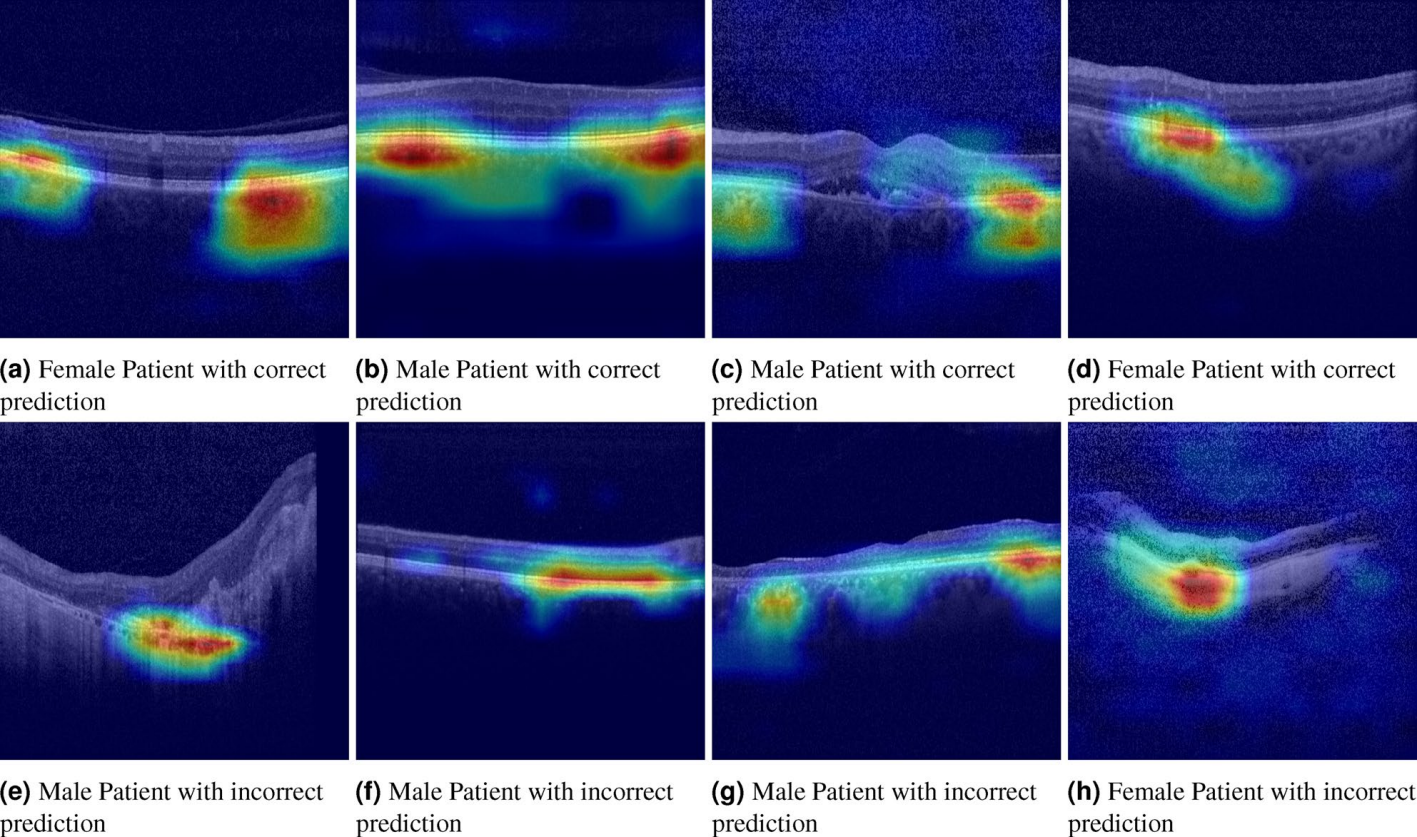
Attention maps of OCT BScans, top row: correct predictions, bottom row: incorrect predictions.

**Figure 9:**
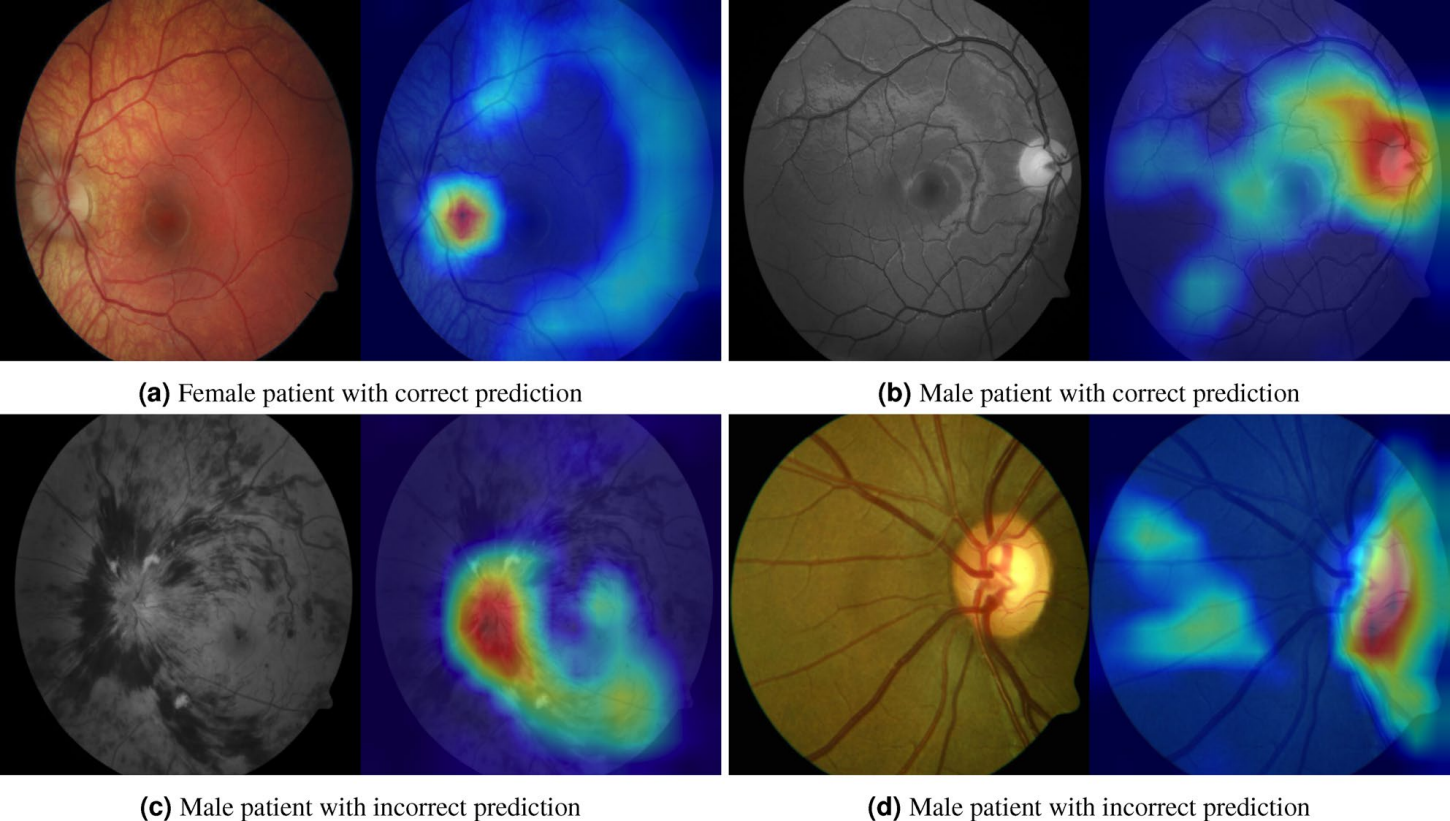
Attention maps of fundus images, top row: correct predictions, bottom row: incorrect predictions.

## Discussion

We here demonstrate that deep learning classifiers can predict gender and age not only based on fundus images, but also based on OCT volume- and on individual Bscans for a broad spectrum of patients and pathologies. This seems independent of image quality, occlusion or other image artifacts typically found in routine clinical care.

We were able to predict gender with an AUC of 0.80 for fundus images, 0.84 for OCT Bscans and 0.90 for OCT Cscan, respectively. Thus, the prediction performance is highest with OCT volume scans, followed by individual OCT Bscans. The least accurate prediction is provided for fundus images. Accordingly the best performance with the lowest mean absolute error on age prediction was found with OCT volume scans, followed by individual OCT Bscans and fundus images, respectively. Considering that the classifier uses the information of 49 images in contrast to only one image in case of Bscans and fundus images, these results are not surprising. The AUC performance for fundus images is lower than the AUC performance described in a previous study for gender and age prediction^10^. In the latter study, color fundus images of mainly diabetic patients of two large biobanks (UK biobank and EyePACS) were used to demonstrate that cardiovascular risk factors, age, smoking status, systolic blood pressure and gender can be abstracted. The AUC performance to predict gender was 0.97. The reason why the performance of that respective model is higher may be manifold. First, a distinct and homogenous patient population was chosen. Second, the images were restricted to central fundus images with a 45-degree field of view. Thus, in all images the optic disc, the vascular arcades and the macula were visible. The patients did not suffer from other ocular diseases such as end stage glaucoma, which lead to significant changes and degeneration of the ocular structures. In particular the optic disc, the peripapillary area, the macula and the visibility of the larger vessels within the posterior pole seem crucial for correct gender and age prediction. The activation maps generated in our study consistently and reliably highlighted those respective regions and our sub analysis on 260 randomly selected predictions demonstrated that without a visible optic disc and macula only random gender prediction is possible. The importance of these regions were also described in Poplin et al.^10^. This may be due to the fact that the fovea of males is smaller than that of females, which can be assessed on fundus images, structural OCT and OCT angiography scans^16–18^. Parameters of the optic nerve head such as RNFL thickness differ too and change during aging^19^. Also vessels undergo significant changes during lifetime and are therefore a good prediction marker for age. Morphology vessel parameters also differ between sexes. Difference in risk and complications of diseases such as diabetes between male and females are partly attributed to respective disparities^20, 21^. Furthermore, retinal and choroidal thickness varies between male and females and the retina and the choroid are significantly thicker in males than in females^16, 19, 22^. This is considered in clinical trials, where the inclusion criteria of central retinal thickness varies between sexes^23^

Despite including patients with a variety of diseases the prediction accuracy for age and gender was very high on OCT volume scans. The prediction accuracy continuously improved with the rate of healthy scans (defined by absence of any of the 11 assessed biomarkers) within the volume scans. With at least 80% healthy scans, the gender prediction increased to an AUC of 0.95. Congruently individual healthy labelled B-scans led to an improved gender and age prediction as well. Interestingly the attention maps highlighted the entire extent of the choroid and indicate that choroidal structures and thickness may be an important differentiator for a correct gender and age prediction. While parameters such as foveal avascular zone, foveal depression and retinal thickness (in particular central retinal thickness) differ between gender^16, 22^, they are not undergoing significant changes as we advance in years. Several choroidal biomarkers have been described including choroidal vessel diameter, vessel diameter index, the choroidal vascular index and choroidal thickness parameters such as choroidal volume and choroidal thickness^24–29^. In particularly choroidal thickness parameters as well as vessel diameter index significantly differs between gender and are correlated with age^16, 29^. Incorrect prediction of gender as well as age on OCT was mainly seen in cases with significantly impaired and degraded choroidal structures, e.g. due to choroidal and outer retinal atrophy or due to invisibility of choroidal structures. The automated biomarker detection further supports this assumption. While the presence of subretinal or intraretinal fluid did not have much impact on correct gender and age prediction, biomarkers such as fibrovascular PED, drusen and particularly geographic atrophy lead to an significant decrease in prediction accuracy. Respective biomarkers are associated with impaired outer retinal and choroidal structures. Of course these results have to be cautiously interpreted. The algorithm only qualitatively assessed the presence or absence of perspective biomarkers without quantitative evaluation. Further multiple biomarker may be present in one singular scan and may further impact and influence the accuracy of the prediction. In healthy scans the best performance was seen in central scans. In case of a present biomarker, however the performance tended to be better in more peripheral scans. Considering that many pathological findings will be more pronounced in the fovea than para- and extrafoveally this finding is coherent.

While there are several studies which evaluated the possibility to assess cardiovascular risk factors based on vessel caliber on fundus images^30^, only one paper so far aimed to extract patient specific information using color fundus images of patients with diabetes. So far no study is available to assess whether these kind of patient specific information can be also extracted from OCT images. While the impact and applicability of the assessment and extraction of cardiovascular risk factors is well established and may help to stratify the risk of cardiovascular events, the ability to do for age and gender is critical for data security and patient privacy. With increasing interest in AI, there is a raising concern about the protection of data privacy^31^. Data protection working party of the European Union specifically list retinal and vein patterns as biometric data that are both unique and identifiable for a given individual. Data anonymization and the risk of re-identification is a major topic and the risk of re-identification of “anonymized” clinical/clinical-trial-data has to be carefully assessed^32^. The robustness of anonymization has to be continuously re-evaluated in the rapidly changing data and AI based data processing environment^32^. In the context of new emerging models and machine learning, continuous re-assessment of the risk of re-identification seems more important than ever before. This is acknowledged by the major regulators such as the European Medicines Agency (EMA) and the Food and Drug Administration (FDA)^32^. The potential of data extraction such as subjects gender and age may therefore lead in the near future to significant changes in the way one saves, stores and shares patient data.

In summary, we here show that accurate gender and age prediction is possible using fundus images as well as OCT scans, irrespective of image quality and retinal pathologies. To the best of our knowledge, this is the first study that compares the prediction of patient specific information from both fundus and OCT imaging. The possibility of extracting so far unknown information using AI may be useful in the future to not only predict obvious patient specific information such as age and gender but also to stratify risk for cardiovascular and neurodegenerative diseases such as Alzheimer and dementia. The automated assessment of these parameters may also help in the future to allow a more precise prediction of disease entities, disease courses and treatment response. Furthermore, our proposed method for sex and age prediction could be used to perform study cohort analysis to compare multiple anonymous studies.

## Data Availability

The datasets generated during and/or analyzed during the current study are not publicly available due to privacy constraints. The data may however be available from the University Hospital Bern subject to local and national ethical approvals.
